# Keystone Veterinary Medical Association

**Published:** 1896-06

**Authors:** J. R. Hart, W. L. Rhoads

**Affiliations:** President; Secretary


### SOCIETY PROCEEDINGS.

### KEYSTONE VETERINARY MEDICAL ASSOCIATION.

THE April meeting was called to order on the evening of the 14th at 8.30 by Dr. Hart, with the following members present: Drs. Hart, Hoskins, H. P. Eves, Chas. Lintz, J. T. McAnulty, F. S. Allen, and W. L. Rhoads.

After a report of the work done by the State Board of Veterinary Examiners Dr. Hoskins, by request, made a report as one of the committee appointed by the Mayor for the selection of a consulting veterinary for the city's staff of meat and milk inspectors. Dr. Allen was then elected to membership by unanimous vote.

The question of society certificates was agitated, and, after a short discussion, Hoskins and Rhoads were appointed by the President a committee of two to report at the next meeting as to cost, etc.

Dr. Chas. Lintz, being the essayist for the evening, read a very interesting paper on "Pleurisy, Acute and Chronic," in which he spoke of several very interesting points on causes, symptoms, complications, sequelæ, and remedies ordinarily used; also those he had found most beneficial. In the first stage (which the practitioner seldom sees unless favorably near at hand) he advocates the use of mustard to the sides and sternum; also, ℞. Quinine, ʒiv; tereb. and acacia pulv., āā ʒvi to ℥iss; ol. lini q. s. ft. f℥xij. Sig. Give ℥j at dose. In the second stage, do away with the mustard and alternate the tereb. with salicylate of soda, every two hours. After five or seven days, iodide of potassium. This paper aroused an earnest discussion, some advocating the use of most powerful heart-stimulants—nitro-glycerine and nux vomica—claiming digitalis was not to be relied upon.

Dr. Hoskins cited case of brown mare, eight years old (driver), weighing 1050 pounds, which at times appeared dull, and had a peculiar throwing of the head, profuse sweating, and at these times a slight incoördination of gait, due to cerebral anæmia, death having resulted from a very short and severe attack of pulmonary apoplexy, which diagnosis was corroborated on autopsy.

The meeting adjourned to reconvene May 12th.

THE May meeting was called to order at 8.30 on the evening of the 12th ult., by President John R. Hart, with the following members of the profession present: Drs. J. R. Hart, F. S. Allen, Charles Lintz, W. Horace Hoskins, H. A. Hackley, W. J. McClellan, and W. L. Rhoads.

Dr. Hoskins, as chairman of the committee appointed at a previous meeting to look up the question of cost, etc., of certificates, made a very favorable report, and, on motion of Dr. Lintz, the committee was instructed to procure a number of these certificates before the next meeting, as at that time a thorough revision of the membership list will be made, and certificates given to those who are at that time in good standing; those in arrears will, by motion of the Society, be expelled from membership, it being deemed advisable to make the separation at this time, as all delinquents have had ample notice and full time in which to settle. The Society, though just ending a most prosperous year, feels that it is being handicapped with its inert members.

Dr. Hoskins, by request, made a report as a member of the State Board of Veterinary Examiners, stating that out of fourteen applicants but three were successful; he said that the examination papers throughout showed that a good primary education was very essential to those who would become veterinarians, and urged that a higher standard be made in the examinations of matriculants by the colleges, if they would at all times graduate men who would be an honor to their alma mater. The next examination will be held by the board June 15th to 16th, inclusive. After July 1, 1896, all applicants for examination must come from three-year schools, naming a number of colleges whose graduates would not be eligible to examination. He also gave Professors Brenton and McEachran, late of the Detroit Veterinary College great credit for their laudable efforts to procure for the students there a three-years' course.

It was moved and seconded that an order be drawn for the payment of rent for the current year.

Dr. Lintz now moved that we complete this, one of the most prosperous, enjoyable, and instructive years in the history of the Association, by a light lunch after our next meeting. This was seconded and unanimously agreed to. The watchword of the K.V.M.A. is live and let live.

The President then appointed Drs. Charles Lintz, F. S. Allen, and W. H. Hoskins, a committee to take this matter in charge.

Dr. Rhoads now read several articles from medical journals ridiculing the germ-theory of disease, and the much-talked-of occult science; this, especially the germ-theory, aroused quite an interest, and the adherents to the convictions of Pasteur were very strong in their belief.

The Society adjourned to reconvene June 9, 1896.

J. R. HART,

President.

W. L. RHOADS,

Secretary.

